# Micellization of Lipopeptides Containing Toll-like Receptor Agonist and Integrin Binding Sequences

**DOI:** 10.1021/acsami.4c18165

**Published:** 2024-12-09

**Authors:** Valeria Castelletto, Lucas R. de Mello, Jani Seitsonen, Ian W. Hamley

**Affiliations:** † School of Chemistry, Food Biosciences and Pharmacy, University of Reading, Whiteknights, Reading RG6 6AD, U.K.; ‡ Nanomicroscopy Center, Aalto University, Puumiehenkuja 2, FIN-02150 Espoo, Finland

**Keywords:** lipopeptides, micelles, aggregation, molecular dynamics, biosurfactants, cytocompatibility

## Abstract

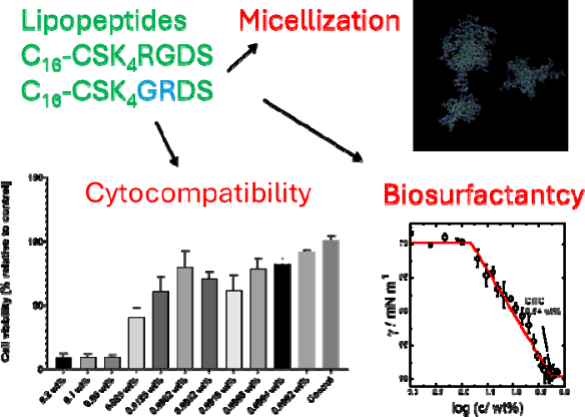

Short bioactive peptide sequences are of great interest in biomaterials development. We investigate the self-assembly of a lipopeptide containing both the highly cationic CSK_4_ toll-like receptor agonist hexapeptide sequence and RGDS integrin-binding motif, i.e., C_16_-CSK_4_RGDS, as well as the control containing a scrambled terminal sequence C_16_-CSK_4_GRDS. Both lipopeptides are found to form micelles, as revealed by small-angle X-ray scattering and cryogenic transmission electron microscopy, and modeled using atomistic molecular dynamics simulations. We carefully examined methods to probe the aggregation of the molecules, i.e. to obtain the critical micelle concentration (CMC). Fluorescent probe assays using 1-anilino-8-naphthalenesulfonate (ANS) reveal low CMC values, 1–2 μM, which contrast with consistent values more than 2 orders of magnitude larger obtained from surface tension and electrical conductivity as well as unexpected UV/vis absorption spectra discontinuities and fluoresccence probe assays using Nile red. The anomalous results obtained from an ANS fluorescence probe are ascribed to the effect of ANS binding to the cationic (lysine and arginine) residues in the lipopeptide, which leads to a conformational change, as shown by circular dichroism, even at low concentrations below the actual CMC. Despite the small change in the peptide sequence (swapping of G and R residues), there is surprisingly a significant difference in the aggregation propensity and association number, both of which are greater for C_16_-CSK_4_GRDS. Both lipopeptides are cytocompatible (with fibroblasts and myoblasts) at low concentration, although cytotoxicity is noted at higher concentration.

## Introduction

Lipopeptides with a remarkable range of applications have been created that incorporate bioactive sequences inspired by natural peptides or designed based on known properties of amino acids or design rules for peptide structures. Such lipopeptides, one type of peptide amphiphile, have been shown to have applications in biomedicine and tissue engineering,^1−7^ as antimicrobial materials,^7−14^ in biocatalysis,^15−18^ and many others.^19^ Lipopeptides can self-assemble into different nanostructures depending on their structure (lipid chain type, length, and peptide sequence) as well as the solution conditions.^2−4,19−24^ A diversity of morphologies have been observed including nanofibrils, nanosheets, nanotubes, vesicles, and micelles. Lipopeptide micelles have been observed as a result of the self-assembly of α-helical peptides,^25,26^ β-sheet-based sequences (although under conditions where disordered conformations are stable)^27,28^ or other short peptide sequences,^15,17,29−37^ conjugates containing intrinsically disordered peptides,^38^ or cyclic lipopeptides.^39−42^

Among short bioactive peptide sequences, the integrin-binding motifs RGD or RGDS have attracted particular attention as adhesion motifs in a vast range of biomaterials research.^43−45^ These minimal cell-adhesive domains are present in extracellular proteins such as fibronectin, fibrinogen, and vitronectin, which all contain integrin ligands.^46−48^ RGD is also presented as an adhesion recognition sequence in other proteins, including laminin and some types of collagen.^49^ These sequences interact with the α_v_β_3_ and α_v_β_5_ receptors and were originally identified as key factors in angiogenesis. Further details and examples are provided elsewhere.^50,51^

Another important bioactive sequence that has been the basis for the development of a series of bioactive lipopeptides used for the development of vaccines and their adjuvants and agents for cancer immunotherapy is the lipid-linked toll-like receptor (TLR) agonist hexapeptide CSK_4_. The lipid glyceryl–cysteine component is derived from bacterial lipopeptides (which stimulate a strong immune response).^52^ Braun’s group developed lipid-linked CSK_4_ as an adjuvant,^53^ based on the N-terminal domain from the murein (peptidoglycan) lipoprotein obtained from the outer cell membrane of *Escherichia coli*.^54,55^ We examined the conformation and self-assembly of PamCSK_4_, Pam_2_CSK_4_, and Pam_3_CSK_4_ (Pam = palmitoyl, C_16_-).^31^ The former two molecules form spherical micelles with the peptide in a disordered conformation, whereas the latter forms flexible wormlike micelles (coexisting with globular structures) with a β-sheet secondary structure and bilayer molecular packing. These structures were later confirmed by molecular dynamics (MD) simulations.^32^ Further details on TLR lipopeptides with examples and applications are discussed elsewhere.^56−59^

The combination of bioactive peptide sequences can confer synergistic or novel properties. Here we investigate the self-assembly and cytocompatibility of a lipopeptide containing both the highly cationic CSK_4_ TLR sequence and RGDS integrin-binding motif, i.e., C_16_-CSK_4_RGDS (PamCSK_4_RGDS), as well as the control containing a scrambled terminal sequence C_16_-CSK_4_GRDS (PamCSK_4_GRDS). The critical micelle concentration (CMC) was obtained from multiple techniques including fluorescence probe assay using an anionic dye or a neutral dye, as well as colligative property measurements of surface tension and electrical conductivity. We also noted a discontinuity in UV/vis spectra (peptide backbone absorbance) that can be associated with the CMC. The formation of micelles was confirmed by high-resolution cryogenic transmission electron microscopy (cryo-TEM) imaging and small-angle X-ray scattering (SAXS) data, the latter providing detailed information on micelle dimensions and structure through analysis of the form factor and intermicellar interactions through structure factor effects. The micelle formation was also modeled via atomistic MD simulations. The combination of SAXS and MD provides unique data on the aggregation propensities (APs) and micelle structures for the two lipopeptides. Finally, the cytocompatibilities of the two lipopeptides were compared using mitochondrial activity (MTT) assays and were found to be good (at sufficiently low concentration) for both fibroblasts and myoblasts.

## Methods

### Materials and Sample Preparation

Lipopeptides C_16_-CSKKKKRGDS (hereafter C_16_-CSK_4_RGDS) and C_16_-CSKKKKGRDS (hereafter C_16_-CSK_4_GRDS) were purchased from Biomatik (Kitchener, Ontario, Canada) and supplied as TFA salts. A second batch of each was purchased from Peptide Protein Research Ltd. (Fareham, United Kingdom). The molar mass measured by ESI-MS for C_16_-CSK_4_RGDS is 1376.5 g mol^–1^ (1374.8 g mol^–1^ expected), and for C_16_-CSK_4_GRDS, it is 1376.7 g mol^–1^ (1374.8 g mol^–1^ expected). The purity (of the Biomatik batch) by high-performance liquid chromatography (HPLC) (0.1% TFA in an acetonitrile–water gradient) is 91.2% for C_16_-CSK_4_RGDS and 91.03% for C_16_-CSK_4_GRDS. The samples from Peptide Protein Research Ltd. have purities of ≥96% from HPLC. Solutions were prepared by dissolution in ultrapure water, leading to pH 2.5 for 1 wt % solutions of each lipopeptide.

### Circular Dichroism (CD) Spectroscopy

Far-UV CD spectra were collected using a Chirascan spectropolarimeter (Applied Photophysics, Leatherhead, U.K.). Spectra were recorded from 180 to 400 nm. Samples were mounted in a quartz cell with detachable windows with a 0.01 or 0.1 nm path length and also were mounted in quartz cuvettes with 1 mm path lengths. The CD signal from the samples was corrected by water background subtraction. The CD spectra were smoothed using the Chirascan Software for data analysis. The residue of the calculation was chosen to oscillate around the average, to avoid artifacts in the smoothed curve. CD data, measured in millidegrees, was normalized to molar ellipticity using the molar concentration of the sample and the cell path length.

### Fluorescence Spectroscopy

To investigate the CMC of both lipopeptides, assays were performed using the fluorescent probes 1-anilino-8-naphthalenesulfonate (ANS) or Nile red. Spectra were recorded using a Cary Eclipse spectrofluorometer with excitation and emission slits fixed at 5 nm and the temperature maintained at 20 °C. ANS is an anionic molecule that does not present a significant fluorescence emission in polar environments but shows enhanced fluorescence emission allied to hypsochromic shifts when inserted into nonpolar environments.^60,61^ For this assay, different concentrations of lipopeptide were solubilized in ultrapure water + 75 μM ANS and placed inside quartz cuvettes and fluorescence was measured with λ_ex_ = 375 nm. Nile red is a neutral lipophilic dye.^62,63^ For this assay, lipopeptides were dissolved in a solution containing 5 μM Nile red, and the excitation wavelength was λ_ex_= 550 nm.

### Surface Tension

The surface tension γ was measured by the Du Noüy ring method using a Krüss K12 processor tensiometer. A dilution series was prepared from a concentrated stock solution. Aliquots of 3 mL of each solution were placed in a 25 mL beaker, and γ was manually measured for each concentration. The solution was left to equilibrate for 10 min before each measurement, before γ was measured from three ring detachments from the surface. The CMC was determined as the concentration at which γ reached a stable value (γ_0_). The slope below the CMC in the plot of γ against log*c* (*c* = molar concentration) was used to calculate the surface area per molecule at the air–water interface, *A*, according to the Langmuir adorption equation for the surface excess:^64,65^
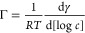
1Here *R* is the gas constant and *T* is the temperature. The surface area per molecule was obtained as
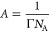
2where *N*_A_ is Avogadro’s number.

### Conductivity

The electrical conductivity, κ, of the solutions was measured using a Zetasizer Nano ZS from Malvern Instruments. An aliquot of 1 mL of the sample was placed inside a disposable folded capillary cell. The conductivity was measured using an applied voltage of 50.0 V. Each data point was recorded using a target of 100 measurements.

### UV/Vis Spectroscopy

UV/vis spectra were measured using a Nanodrop spectrophotometer. For each experiment, an aliquot of 5 μL of peptide solution was placed on the Nanodrop platform, and the UV/vis spectra were measured for wavelengths in the range 190–850 nm. Spectra were corrected using a water background.

### Cryo-TEM

Imaging was carried out using a field-emission cryogenic transmission electron microscope (JEOL JEM-3200FSC), operating at 200 kV. Images were taken in bright-field mode using zero loss energy filtering (omega type) with a slit width of 20 eV. Micrographs were recorded using a Gatan Ultrascan 4000 CCD camera. The specimen temperature was maintained at −187 °C during the imaging. Vitrified specimens were prepared using an automated FEI Vitrobot device using Quantifoil 3.5/1 holey carbon copper grids with a hole size of 3.5 μm. Just prior to use, grids were plasma-cleaned using a Gatan Solarus 9500 plasma cleaner and then transferred to the environmental chamber of a FEI Vitrobot at room temperature and 100% humidity. Thereafter, 3 μL of sample solution was applied on the grid, and it was blotted twice for 5 s and then vitrified in a 1/1 mixture of liquid ethane and propane at a temperature of −180 °C. The grids with vitrified sample solution were maintained at liquid-nitrogen temperature and then cryo-transferred to the microscope.

### SAXS

SAXS experiments were performed on beamline B21^66^ at Diamond (Didcot, U.K.). The sample solutions were loaded into the 96-well plate of an EMBL BioSAXS robot and then injected via an automated sample exchanger into a quartz capillary (1.8 mm internal diameter) in the X-ray beam. The quartz capillary was enclosed in a vacuum chamber to avoid parasitic scattering. After the sample was injected into the capillary and reached the X-ray beam, the flow was stopped during the SAXS data acquisition. Beamline B21 operates with a fixed camera length (3.9m) and fixed energy (12.4 keV). The images were captured using a Pilatus 2 M detector. Data processing was performed using dedicated beamline software *ScÅtter*. Additional experiments were performed using the BioSAXS setup on BM29 at the ESRF (Grenoble, France).^67^ A few microliters of samples were injected into a quartz capillary (1.8 mm internal diameter) in the X-ray beam. The flow of sample was stopped during the SAXS data acquisition. The *q* range was 0.005–0.48 Å^–1^, with wavelength λ = 1.03 Å and a sample–detector distance of 2867 mm. The images were obtained using a Pilatus 3-2M detector. Data processing (background subtraction and radial averaging) was performed using software *ISPyB*.

### MD Simulations

MD simulations were performed using *GROMACS*(68) (versions 2023.2 and 2020.1-Ubuntu-2020.1-1). Molecules of each of the two lipopeptides were packed using *PACKMOL*(69) into spherical micelles with association numbers in the range *p* = 22–60 and a range of charge states corresponding to charged or uncharged arginine, lysine, aspartic acid residues, or C-terminus. The influence of the effect of the presence or absence of hydrogen atoms was also explored in some simulations. The lipopeptide structures were generated using UCSF Chimera. Simulations were performed using the CHARMM27 force field^70,71^ using the included force-field parameters for C_16_ (palmitoyl) chains, which were manually adapted to build the lipid–peptide linking unit. The micelles were placed into simulation boxes (cubes) of length 20 nm, and systems were solvated using spc216 water. Each system was neutralized using a matching number of Cl^–^ counterions. After energy minimization and 100 ps relaxation stages in the NVT and NPT ensembles, the final simulations were carried out in the NPT ensemble using a leapfrog integrator with steps of 2 fs up to 4 or 10 ns depending on the equilibration of the system [as monitored by the lipopeptide radius of gyration (*R*_g_) or atomic root-mean-square deviation (RMSD)]. The temperature was maintained at 300 K using the velocity-rescale (modified Berendsen) thermostat^72^ with a coupling constant of 10 steps. The pressure was maintained at 1 bar using the Parrinello–Rahman barostat,^73^ and periodic boundary conditions were applied in all three dimensions. The particle mesh Ewald scheme^74,75^ was used for long-range electrostatics. Bonds were constrained using the LINCS algorithm,^76^ and the Verlet cutoff scheme^77^ was used. Coulomb and van der Waals cutoffs were 1.0 nm.

### Cytotoxicity Assays

Cytotoxicity was investigated using an assay with 3-(4,5-dimethylthiazol-2-yl)-2,5-diphenyltetrazolium bromide (MTT) (Sigma-Aldrich, U.K.). Initially, L929 murine fibroblasts and C2C12 murine myoblasts were maintained in Dulbecco’s modified Eagle’s medium (DMEM), supplemented with 10% fetal bovine serum (FBS), glutamine, and penicillin–streptomycin, with the reagents being purchased from Thermo Fisher Scientific (U.K.). Cells were seeded at a confluence of 5 × 10^3^ cells in a volume of 100 μL well^–1^ into 96-well plates and incubated for 72 h in DMEM (10% FBS, 1% glutamine, and 1% penicillin–streptomycin). After incubation, cells were incubated for 4 h in DMEM without serum + 0.5 mg mL^–1^ MTT. The resulting formazan crystals were dissolved using 100 μL of DMSO for 30 min at 37 °C, protected from light. The resulting absorbances were read at 570 nm, and the data were subsequently analyzed using *GraphPad Prism* software.

## Results and Discussion

We first investigated potential aggregation of the two lipopeptides C_16_-CSK_4_RGDS and C_16_-CSK_4_GRDS. To locate possible CMC values of the lipopeptides, we performed fluorescence assays in the presence of ANS, an anionic dye molecule that presents negligible fluorescence in water or polar environments. However, when present in a nonpolar environment, there is a significant enhancement in fluorescence emission, thus enabling the use of ANS as a tool for tracking aggregation of proteins, peptides, and other biomolecules.^61,78^ The enhancement in fluorescence is only one of the possible outcomes of the interaction between ANS and peptides/proteins because when ANS binds to sites containing arginine or lysine, the spectra also present hypsochromic shifts, most notably a blue shift due to ion pairing between the cationic groups and the sulfonate group of ANS, leading to a change in intermolecular charge-transfer rate constant upon excitation at a wavelength λ_ex_ = 375 nm, with a more significant blue shift in the presence of arginine.^79^

**Figure 1:**
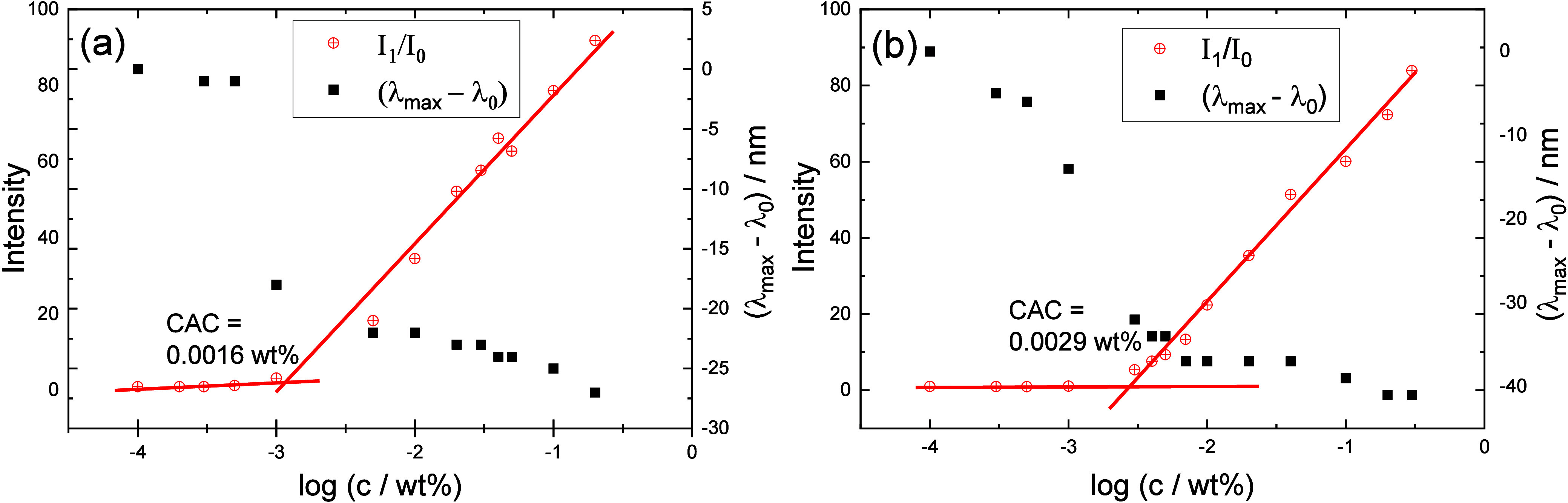
Concentration-dependent ANS fluorescence intensity (left-hand axes, *I*_1_/*I*_0_, relative to the sample without lipopeptide) and fluorescence maximum wavelength shift (relative to the sample without lipopeptide) (right-hand axes) to determine CMC for (a) C_16_-CSK_4_RGDS and (b) C_16_-CSK_4_GRDS.

Concentration-dependent fluorescence data for both lipopeptides are presented in Figure 1, analyzing both the wavelength shift and fluorescence intensity presented as the ratio *I*_1_/*I*_0_, where *I*_0_ is the initial fluorescence maxima of ANS in water and *I*_1_ is the maximum ANS fluorescence intensity in the presence of lipopeptide. The original ANS fluorescence spectra are shown in Figure S1. The data show that both lipopeptides have distinct regimes of fluorescence, which are due to sequestration of ANS in a hydrophobic environment at high concentration, resulting in an enhancement of the fluorescence emission. The CMC was determined from discontinuities in both fluorescence and intensity (which provide consistent values), resulting in values of 0.0016 ± 0.001 wt % for C_16_-CSK_4_RGDS and 0.0029 ± 0.001 wt % for C_16_-CSK_4_GRDS (Figure 1). These values are the same within uncertainty and are equivalent to 1–2 μM. The spectra show a notable blueshift in the spectra of ANS in the presence of both lipopeptides, indicating that the fluorescent probe is in contact with the arginine and lysine residues inside the aggregates, especially for C_16_-CSK_4_GRDS due to the observed larger blue shift in comparison with C_16_-CSK_4_RGDS (Figure 1).

**Figure 2:**
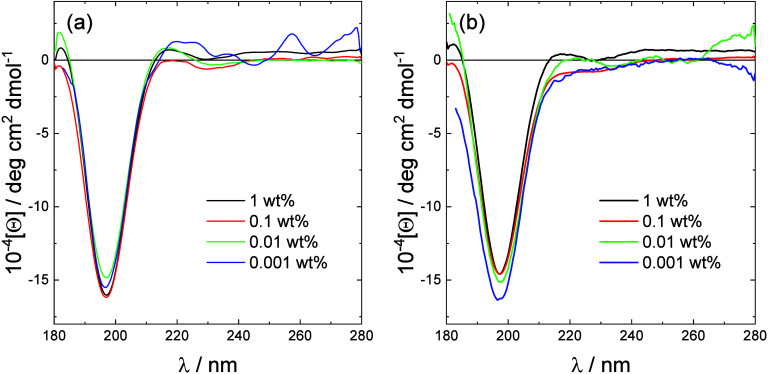
CD spectra for the two lipopeptides at the concentrations shown: (a) C_16_-CSK_4_RGDS; (b) C_16_-CSK_4_GRDS.

The secondary structure of the lipopeptides was probed using CD spectroscopy. Spectra are shown in Figure 2 and show that both lipopeptides adopt a disordered conformation over the concentration range examined, which spans the CMC values from ANS fluorescence (and from other measurements, to be discussed shortly). The observation that the peptides have a disordered conformation is consistent with the formation of micelles, as described below. The CD data show that the peptide conformation does not change during lipopeptide aggregation into micelles.

**Figure 3:**
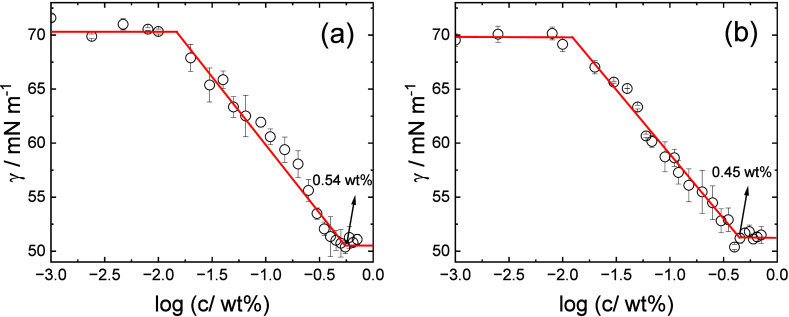
Concentration-dependent surface tension data for (a) C_16_-CSK_4_RGDS and (b) C_16_-CSK_4_GRDS.

Surface tension measurements were used to examine the biosurfactant properties of the two lipopeptides and to estimate CMC values. The data shown in Figure 3 show that, for C_16_-CSK_4_RGDS, the CMC (the concentration at which the surface tension levels off) is 0.54 ± 0.05 wt %, whereas for C_16_-CSK_4_GRDS, it is lower at 0.45 ± 0.05 wt %. The lower CMC value suggests a slightly higher AP for C_16_-CSK_4_GRDS, which is supported by MD simulations discussed below. The limiting surface tensions for both molecules are γ = 51 mN m^–1^.

The slopes of the linear parts of the Gibbs adsorption isotherms (below the CMC) are −10.8 ± 0.5 mN m^–1^ for C_16_-CSK_4_RGDS and −13.9 ± 0.9 mN m^–1^ for C_16_-CSK_4_GRDS. These lead via eq 1 to values of surface excess Γ = 4.42 × 10^–6^ mol m^–2^ for C_16_-CSK_4_RGDS and Γ = 5.72 × 10^–6^ mol m^–2^ for C_16_-CSK_4_GRDS, respectively. From eq 2, this leads to areas per molecule *A* = 37.5 and 29.0 Å^2^, respectively. Considering also the calculated surface area of a micelle core *a*_mic_ = 4π*R*^2^ (and taking *R* = *R*_i_ = 14.0 Å for 2 wt % solutions of either lipopeptide; Table S1), the association number can be estimated as *p* = *a*_mic_/*A* = 66 for C_16_-CSK_4_RGDS and *p* = *a*_mic_/*A* = 85 for C_16_-CSK_4_GRDS. These estimates are significantly larger than those obtained from the SAXS data and MD simulation results presented below.

**Figure 4:**
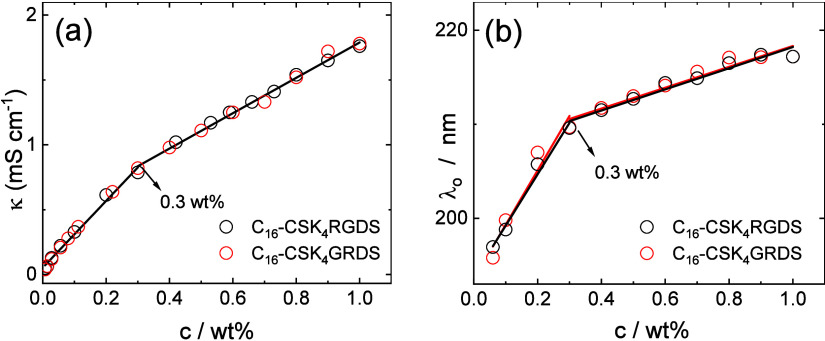
Concentration dependence of the (a) electrical conductivity and (b) UV/vis absorption peak maximum wavelength for the two lipopeptides.

Because there is a difference in the apparent CMC from ANS fluorescence and surface tensiometry, we measured the electrical conductivity κ as an additional colligative property measurement. This shows a discontinuity at the CMC for ionic surfactants due to the association of charged molecules into micelles (with associated counterions).^64,65,80^ The data are shown in Figure 4a, and for both lipopeptides, the conductivity data show similar behavior (as expected given the identical charge on the molecules), with a discontinuity in the slope indicating CMC = 0.3 ± 0.05 wt %, which is in good agreement with the value from surface tension measurements (Figure 3). We also unexpectedly observed discontinuous behavior in the UV/vis absorption spectra for lipopeptide solutions around the CMC. The concentration dependence of the position of the peak maximum shows a change in slope close to the CMC obtained from surface tension and electrical conductivity measurements, providing a further indication for CMC = 0.3 ± 0.05 wt % for both C_16_-CSK_4_RGDS and C_16_-CSK_4_GRDS. The original spectra are shown in Figure S2 and show the pronounced shift in the peak position, with a double peak structure at the highest concentrations studied (i.e., 0.8–1 wt %). The peak in the UV/vis spectra in the range 200–220 nm is due to the peptide backbone (there are no aromatic residues in either lipopeptide).^81,82^ Because the CD spectra for the lipopeptides show no concentration-dependent trends across the CMC (Figure 2), i.e., there is no evidence for concentration-dependent backbone conformation changes, the changes in the UV/vis absorption spectrum are ascribed to the aggregation-driven development of turbidity above the CMC, with this being another colligative property used to locate the CMC.^64,65,80^

**Figure 5:**
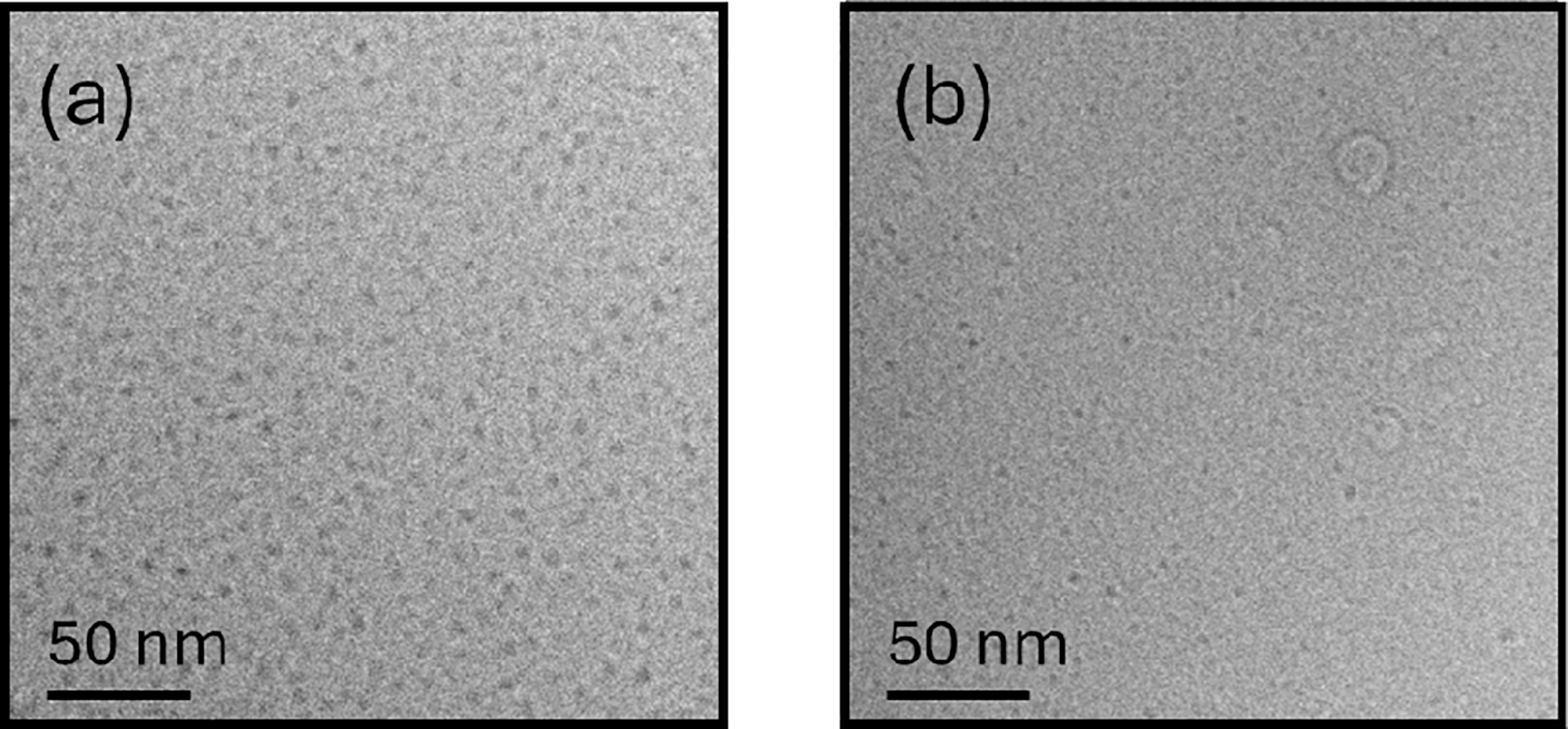
Cryo-TEM images from 0.5 wt % aqueous solutions of (a) C_16_-CSK_4_RGDS and (b) C_16_-CSK_4_GRDS.

Cryo-TEM and SAXS were used to comprehensively examine the self-assembled structure of the lipopeptides in aqueous solution. Cryo-TEM images in Figure 5 clearly show the presence of globular micelles with a diameter of approximately 4 nm for both lipopeptides (for C_16_-CSK_4_GRDS, a small fraction of small vesicle-like structures was also noted in the cryo-TEM images; some can be seen in Figure 5b).

**Figure 6:**
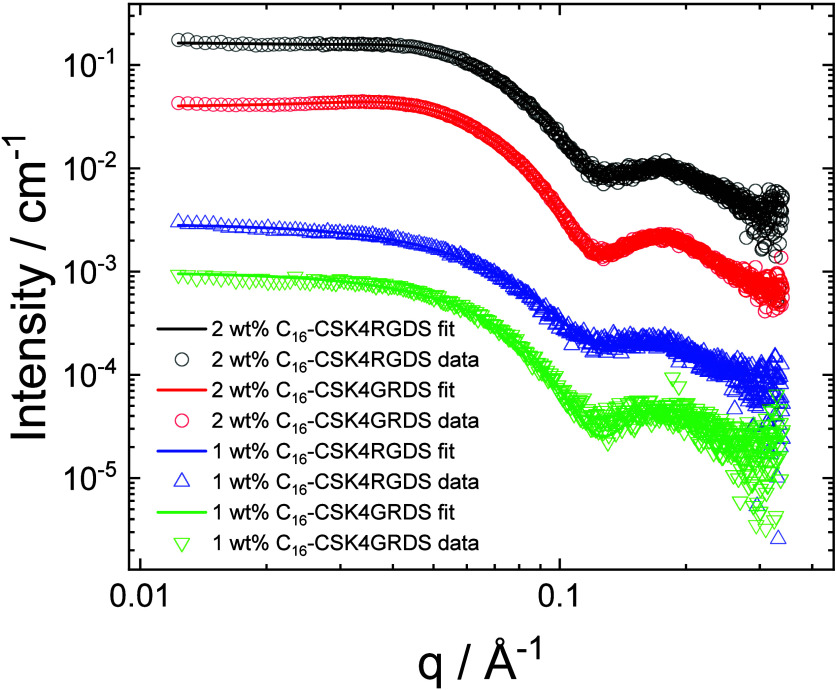
SAXS data from 1 or 2 wt % aqueous solutions of the two lipopeptides. Open symbols are measured data, and the solid lines are fits as described in the text (fitting parameters in Table S1). For ease of visualization, only every 5th data point is shown and the data for 2 wt % C_16_-CSK_4_GRDS are offset by division by 5, those for 1 wt % C_16_-CSK_4_RGDS by 25, and those for 1 wt % C_16_-CSK_4_GRDS by 125.

The SAXS data for the two lipopeptides are shown in Figure 6. The SAXS data for C_16_-CSK_4_GRDS can be fitted using a form factor of core–shell spheres, used to fit the SAXS data from spherical micelles. The fit parameters are listed in Table S1. For the higher concentration samples, the notable decrease in scattering at low *q* (with the development of a broad maximum around *q* = 0.04 Å^–1^) is due to the development of a structure factor, which could be accounted for using a hard-sphere structure factor (the simplest applicable model, with only two fit parameters). The SAXS data for C_16_-CSK_4_RGDS show important differences compared to C_16_-CSK_4_GRDS, and fitting the data with physically meaningful parameters required consideration of a contribution due to unaggregated molecules (monomers), represented as generalized Gaussian coils (i.e., Gaussian coils, allowing for expansion/contraction through variation of the Flory exponent). Again, structure factor effects were noted for the data for the 2 wt % solution. The micelles for both lipopeptides are characterized by an outer radius *R*_0_ = 30 Å and an inner radius *R*_i_ = 14–16 Å. A feature of the data is the low shell–core scattering contrast values compared to those for the well-defined micelles of C_16_-WKK and C_16_-YKK (and homologues with d-Trp and d-Tyr) recently studied by our group.^83^ The reduced aggregation tendency of C_16_-CSK_4_RGDS (i.e., the presence of micelles coexisting with monomers) is indicated both by the shape of the overall scattering profile and also by the following analysis of the forward scattering.

The micelle association number can be obtained in a model-independent fashion from the forward scattering intensity of the SAXS data.^84,85^ The measured SAXS data presented here is in absolute units (cm^–1^), and the forward scattering (at *q* = 0) can be written as^86^
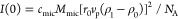
3Here *c*_mic_ is the concentration of micelles, *M*_mic_ is the micelle molar mass, *r*_0_ is the classical electron radius (0.28179 × 10^–12^ cm e^–1^), *v*_p_ is the partial specific volume, and ρ_l_ and ρ_0_ represent the lipopeptide and solvent (water) electron densities. Here we wish to obtain the micelle molar mass and hence *p*. Rearranging eq 3 gives
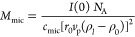
4

For either lipopeptide, using the equation due to Tanford for the volume per lipid chain,^87^*v*_l_ = 27.4 + 26.9*n* (where *n* is the number of carbons in the lipid chain excluding the terminal CH_3_ group, i.e., *n* = 15, and *v*_l_ is in units of Å^3^) gives *v*_l_ = 430.9 Å^3^. The tail contains 129 electrons in this volume, i.e., the electron density is ρ_l_= 0.299 e Å ^–3^ (which is close to the expected electron density for methylene groups in alkyl chains^88,89^). The electron density of water is taken as ρ_0_ = 0.333 e Å^–3^. Taking a concentration *c*_mic_ = 1 wt % (0.01 g cm^–3^) and with *v*_p_ = 259.5 cm^3^ mol^–1^/225 g mol^–1^ = 1.15 cm^3^ g^–1^ and using *I*(0) = 0.11 cm^–1^ (for the data for a 1 wt % solution of C_16_-CSK_4_GRDS in Figure 6) leads to *M*_mic_ = 5.46 × 10^4^ g mol^–1^, i.e., *p* = *M*_mic_/*M*_mol_ = 40 (here *M*_mol_ = 1376.7 is the molar mass of C_16_-CSK_4_GRDS). For C_16_-CSK_4_RGDS, the significantly lower forward scattering *I*(0) = 0.06 cm^–1^ (Figure 6) leads to a lower *p* = 22; however, this should be considered only an estimate due to the above-mentioned significant presence of monomers in the solution of this lipopeptide.

**Figure 7:**
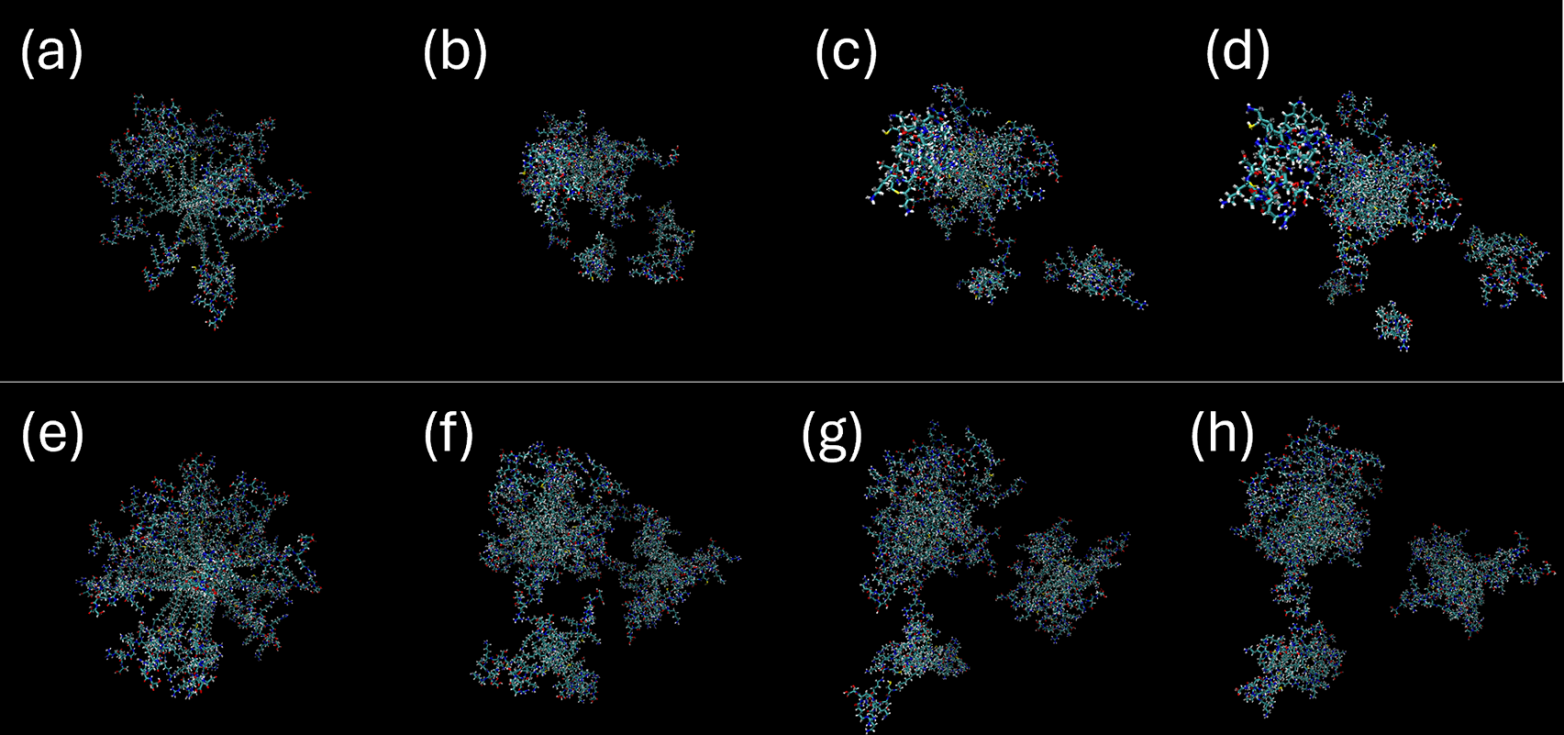
Images of micelles obtained from MD simulations. Top: C_16_-CSK_4_RGDS with *p* = 22 (charge of 2+ per molecule) at *t* = (a) 0, (b) 1, (c) 2.5, and (d) 4 ns. Bottom: C_16_-CSK_4_GRDS with *p* = 40 (charge of 2+ per molecule) at *t* = (e) 0, (f) 1, (g) 2.5, and (h) 4 ns.

The structure and properties of micelles of the two lipopeptides were examined and compared using atomistic MD simulations. The simulations revealed splitting of the initial spherical configurations into smaller oligomeric clusters with 5–15 molecules, as exemplified by the images in Figure 7. An extensive range of simulations were performed for a range of association numbers and molecular charges to examine this in more detail because the stability of the micelles can be influenced by both of these quantities. For a range of association numbers *p* = 20–60 (for both molecules) and charges per molecule from +4 to +2 (allowing for the possibility for neutral charged residues and/or C-terminus), the same phenomenon of splitting of the initial configurations into smaller “micelles” (oligomer clusters) was observed, as exemplified by the frames shown in Figure 7. This appears to occur via a fission-type process. This tendency was more noticeable in simulations for C_16_-CSK_4_RGDS. Additional frames showing this breakup of micelles into smaller aggregates for systems with *p* = 60, for example, are shown in Figures S3 and S4. The calculated properties, for example, solvent-accessible surface area (SASA) and solvation-related properties (as well as the radius of gyration, *R*_g_, of the micelle and atom position RMSDs), were found to plateau during the simulation length (4–10 ns), pointing to equilibrium being reached (and no further dissociation of aggregates was observed). This is exemplified by the data in Figure S5. The AP may be calculated as the ratio of the initial SASA to the final value.^90^ For the data in Figure S5a for C_16_-CSK_4_RGDS, AP = 1.40 ± 0.01, and from Figure S5b, for C_16_-CSK_4_GRDS, AP = 1.52 ± 0.01. It is evident that the AP for C_16_-CSK_4_GRDS is significantly higher. These values of AP correspond to quite weakly aggregating systems,^90^ which along with the MD trajectories showing stable small aggregates, indicates that the molecules have quite a low propensity to aggregate into small micelles. This is consistent with the SAXS data analysis, which shows low shell–core contrast (and considerable polydispersity in radius in some cases) along with the presence of a notable contribution from monomers for C_16_-CSK_4_RGDS.

**Figure 8:**
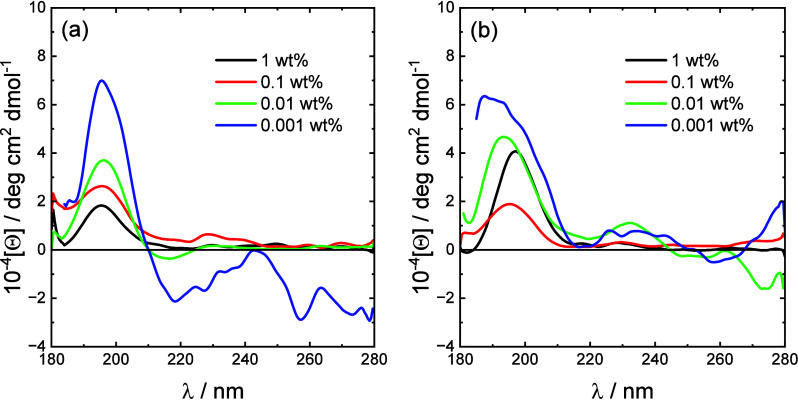
Difference CD spectra [lipopeptide – (lipopeptide + 0.002 wt % ANS)] for the two lipopeptides at the lipopeptide concentrations shown: (a) C_16_-CSK_4_RGDS; (b) C_16_-CSK_4_GRDS.

Our studies of aggregation reveal a substantial difference in the apparent CMC from ANS fluorescence probe measurements and the other methods (surface tensiometry, electrical conductivity, and UV/vis absorption) employed. The former method gives much lower values, CMC = 0.0016 ± 0.001 wt % for C_16_-CSK_4_RGDS and CMC = 0.0029 ± 0.001 wt % for C_16_-CSK_4_GRDS, compared to values from surface tension CMC = 0.54 ± 0.02 wt % for C_16_-CSK_4_RGDS and CMC = 0.45 ± 0.02 wt % for C_16_-CSK_4_GRDS. We sought to examine this difference in more detail. We examined whether the ANS probe molecule can influence the conformation of the peptide sequences, inspired by prior work that shows binding of the dye to proteins due to interactions between anionic ANS and oppositely charged lysine or arginine residues, as well as changes in the CD spectra of poly arginine in the presence of ANS indicating an induced coil–helix transition.^79^ We measured CD spectra, considering that ANS itself is nonchiral and the signal arises only from the peptide sequence in the lipopeptide. Difference CD spectra [lipopeptide – (lipopeptide + 0.002 wt % ANS)] are plotted in Figure 8 (the original spectra are presented in Figures S6 and S7). While there is some noise in the spectra at the lowest concentration (0.0001 wt %, especially for C_16_-CSK_4_RGDS), for both samples for all concentrations, the difference spectra show a positive maximum near 200 nm, along with a shallow minimum near 215 nm. These are signatures of β-sheet conformation.^91−94^ These results suggest that ANS binding can change the conformation of the lipopeptides, even at very low concentration. Therefore, ANS cannot be considered a nonperturbative probe of aggregation due to its binding to the arginine/lysine residues in the peptide sequences, and we ascribe the apparent CMC from this technique as reflecting the binding of ANS to unaggregated molecules (leading to a conformational change), even below the actual CMC, which is detected by nonperturbative colligative property measurements including surface tension. As a further check on this, we performed additional fluorescence probe measurements of CMC using Nile red, which is a neutral lipophilic dye, in contrast to anionic ANS. The data shown in Figure S8 (original spectra in Figure S9) provide values of CMC = 0.68 ± 0.05 wt % for C_16_-CSK_4_RGDS and CMC = 0.38 ± 0.01 wt % for C_16_-CSK_4_GRDS. These are similar to the values from the surface tension, electrical conductivity, and UV/vis absorption spectra maxima and indicate that Nile red acts a nonperturbative probe, in contrast to ANS, which undergoes electrostatic binding to the cationic residues in the lipopeptides and changes their conformation.

**Figure 9:**
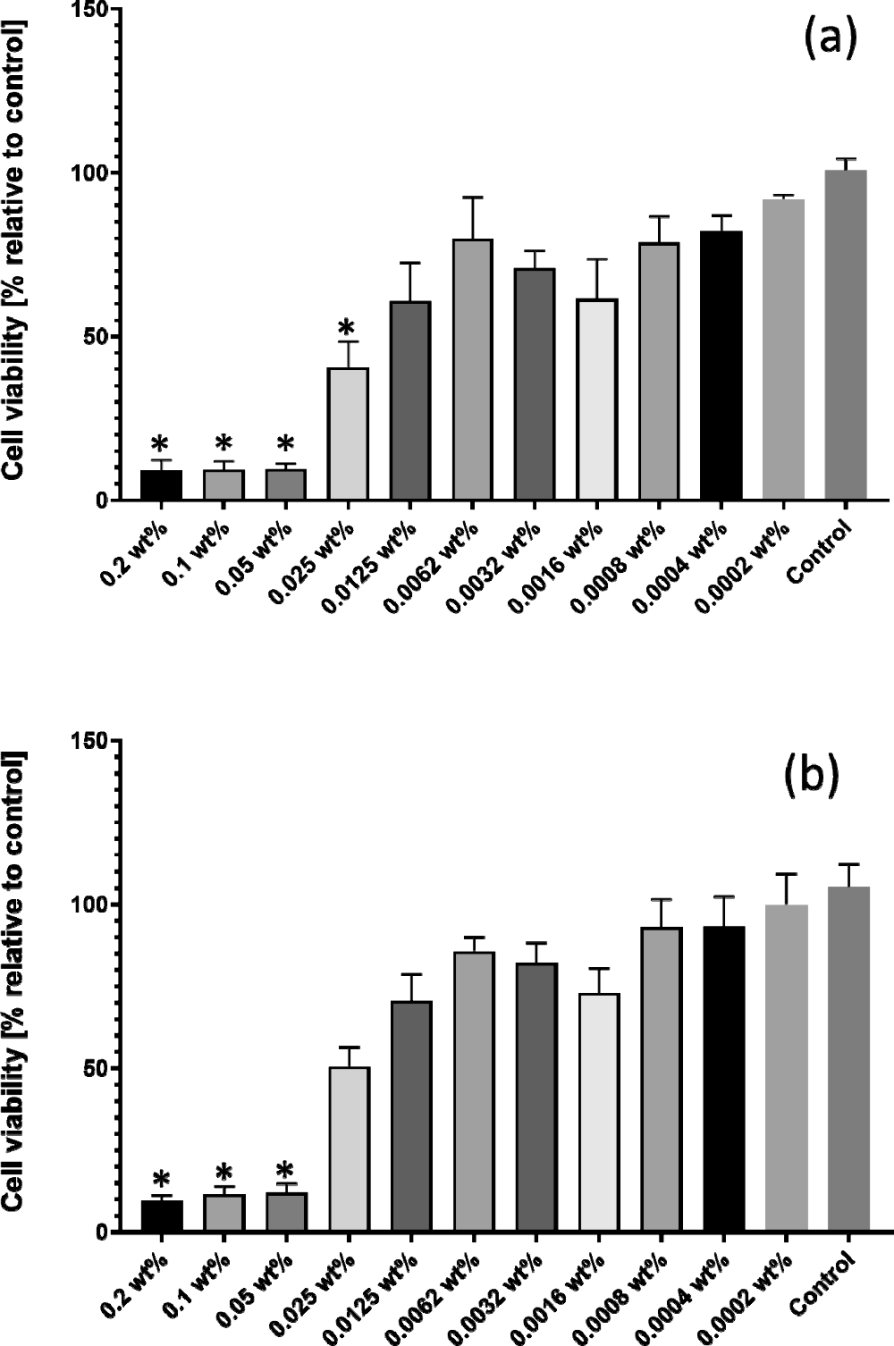
Cell viability after 72 h for L929 fibroblasts from MTT assays for (a) C_16_-CSK_4_RGDS and (b) C_16_-CSK_4_GRDS. *p ≤ 0.05.

The C_16_-CSK_4_RGDS lipopeptide is designed to incorporate the bioactive integrin-binding sequence RGDS (as well as bioactive CSK_4_), whereas C_16_-CSK_4_GRDS contains a scrambled C-terminal tetrapeptide sequence. The cytocompatibility of both lipopeptides was examined using MTT assays to track the effect of the two lipopeptides on the metabolic activity of two types of cells, C2C12 myoblasts and L929 fibroblasts. The reduction of MTT occurs inside the mitochondria and is an indirect indicator of cell health, due to the importance of energy generation and many other processes that occur inside the mitochondrial complex to maintain cell homeostasis.^95,96^Figure 9 shows the MTT results obtained after incubating L929 cells with both lipopeptides for 72 h. The data show that C_16_-CSK_4_RGDS was tolerated for concentrations below 0.0125 wt %, while C_16_-CSK_4_GRDS only exhibits a significant reduction in cell viability over 0.025 wt %. Similar results were observed with C2C12 cells (Figure S10), with both peptides only presenting significant loss of cell viability for concentrations of 0.05 wt % or above. This is significantly below the CMC from surface tension and electrical conductivity measurements, so there does not appear to be any correlation between cytotoxicity and self-assembly. The cytotoxicity observed at higher concentration is presumably due to the presence of the cationic residues (tetra-lysine and arginine) in the peptide sequence, which can interact with zwitterionic cell membranes.

## Conclusions

We have critically examined the micellization properties of lipopeptides C_16_-CSK_4_RGDS and C_16_-CSK_4_GRDS. The ANS fluorescence probe assay shows discontinuities at low concentrations (0.0016–0.0029 wt %, 1–2 mM), 2 orders of magnitude lower than the values obtained from surface tension, electrical conductivity, and Nile red fluorescence spectra measurements (0.3–0.7 wt %), which are believed to correspond to the “true” CMC. The ANS probe measurements result from binding of the anionic dye to lysine and arginine residues in the peptide, and we show through CD spectroscopy that this leads to peptide conformational changes. This “pre-transition” occurs below the onset of aggregation, i.e., the CMC detected by colligative property measurements. We also unexpectedly found that UV/vis spectra (peptide backbone absorbance peak) show a discontinuity at the same concentration as the CMC from surface tension and electrical conductivity. We are not aware of the prior use of this method to detect aggregation, and it may be a useful assay in the future for other peptide-based aggregators. The change in UV/vis spectra is ascribed to the development of turbidity upon aggregation rather than backbone conformational changes because CD spectroscopy indicates no conformational changes across the CMC. We previously noted the difference between the apparent CMC from consistent surface tension and electrical conductivity measurements^83^ compared to ANS fluorescence assays^13^ for lysine-based lipopeptides C_16_-YKK and C_16_-WKK (and analogues with d-lysine), and here we have proposed a possible explanation for this effect due to ANS-binding-induced peptide conformation changes. We conclude that ANS fluorescence measurements may significantly underestimate the CMC for micelle-forming cationic lipopeptides.

A detailed analysis of the micelle structure and aggregation tendency of C_16_-CSK_4_RGDS and C_16_-CSK_4_GRDS from SAXS and MD simulations reveals an unexpected difference for the two molecules, even though they are homologous with just one swap of the G and R residues. The AP (and association number) for C_16_-CSK_4_GRDS is significantly higher than that for C_16_-CSK_4_RGDS. This presumably reflects the role of the uncharged glycine spacer, which separates like-charged cationic residues in C_16_-CSK_4_GRDS (so that R and D are adjacent and salt bridges may form) or oppositely charged residues in C_16_-CSK_4_RGDS. The higher AP of C_16_-CSK_4_GRDS leads to a lower CMC, as well as a higher association number (from SAXS and MD analysis).

The limiting surface tension for both molecules γ = 51 mN m^–1^ is high compared to the values for natural lipopeptides derived from bacteria, which have cyclic peptide headgroups, for which, typically, γ = 20–30 mN m^–1^.^97−104^ Zhang et al. reported lower limiting values in the range γ = 36.7–44.4 mN m^–1^ for linear lipopeptides bearing di- or triglycine peptides.^105^ We reported values γ = 45.6–49.6 mN m^–1^ for a series of lipopeptides with lysine-based tripeptide headgroups.^83^ Low surface tensions γ = 32.4–34.6 mN m^–1^ were noted for lysine-based surfactants bearing two lipid chains connected by an ε-amino lysine-based linker that form micelles (even lower values were observed for systems with longer alkyl chains that form vesicles).^35^ Thus, C_16_-CSK_4_RGDS and C_16_-CSK_4_GRDS show some biosurfactancy properties, but there is scope to further improve this compared to other lipopeptides, if this is the desired application, by suitable sequence modification.

The designed lipopeptides show excellent cell viability for both L929 fibroblasts and C2C12 myoblasts for concentrations below 0.05 wt %. The former are models for cells in connective tissue (e.g., skin), while the latter have been used as model cells that can differentiate into myoblasts. In our previous study on RGDS-containing lipopeptides C_*n*_-GGGRGDS or C_*n*_-WGGRGDS (*n* = 14 or 16), we discussed possible applications for RGDS-based lipopeptides, which show good compatibility (in solution and as hydrogels) for C2C12 cells as scaffolds for cultured meat.^106^ For these RGDS-based lipopeptides, cytocompatibility for C2C12 and L929 was good at 0.01 wt % but cytotoxicity was observed at 0.1 wt %,^106^ although this was less pronounced for the myoblasts. The lysine-based lipopeptides C_16_-YKK and C_16_-WKK (and analogues with d-lysine) show cytotoxicity to L929 fibroblasts in general above 0.01–0.05 wt %,^13^ similar to the observations here, although these lipopeptides have fewer cationic residues. The promising initial cytocompatibility data presented here also point toward such potential applications in tissue engineering or cell culture.

## Supporting Information Available

Fluorescence and UV/vis spectra, Nile red fluorescence CMC assay data, images of micelles from MD simulations, plots of calculated SASA, Δ*G*(solv), density and volume from MD simulations, difference CD spectra from ANS binding studies, and MTT cytocompatibility data for C2C12 myoblasts (PDF)
