# Supplementary material for: Micellization of Lipopeptides Containing Toll-like Receptor Agonist and Integrin Binding Sequences
Source: ACS Appl Mater Interfaces. 2024 Dec 9;16(50):68713–23. doi: 10.1021/acsami.4c18165 (PMC11660038; doi:10.1021/acsami.4c18165)
Supplement: Supplementary file 1 — am4c18165_si_001.pdf [file am4c18165_si_001.pdf]

**Supporting Information**

# Micellization of Lipopeptides Containing Toll-Like Receptor Agonist and Integrin Binding Sequences

Valeria Castelletto,[1] Lucas R. de Mello, [1] Jani Seitsonen,[2] Ian W. Hamley[1,*]

[1] *School of Chemistry, Food Biosciences and Pharmacy, University of Reading, Whiteknights, Reading RG6 6AD, U.K.*

[2] *Nanomicroscopy Center, Aalto University, Puumiehenkuja 2, FIN-02150 Espoo, Finland*

* Author for correspondence: I.W.Hamley@reading.ac.uk

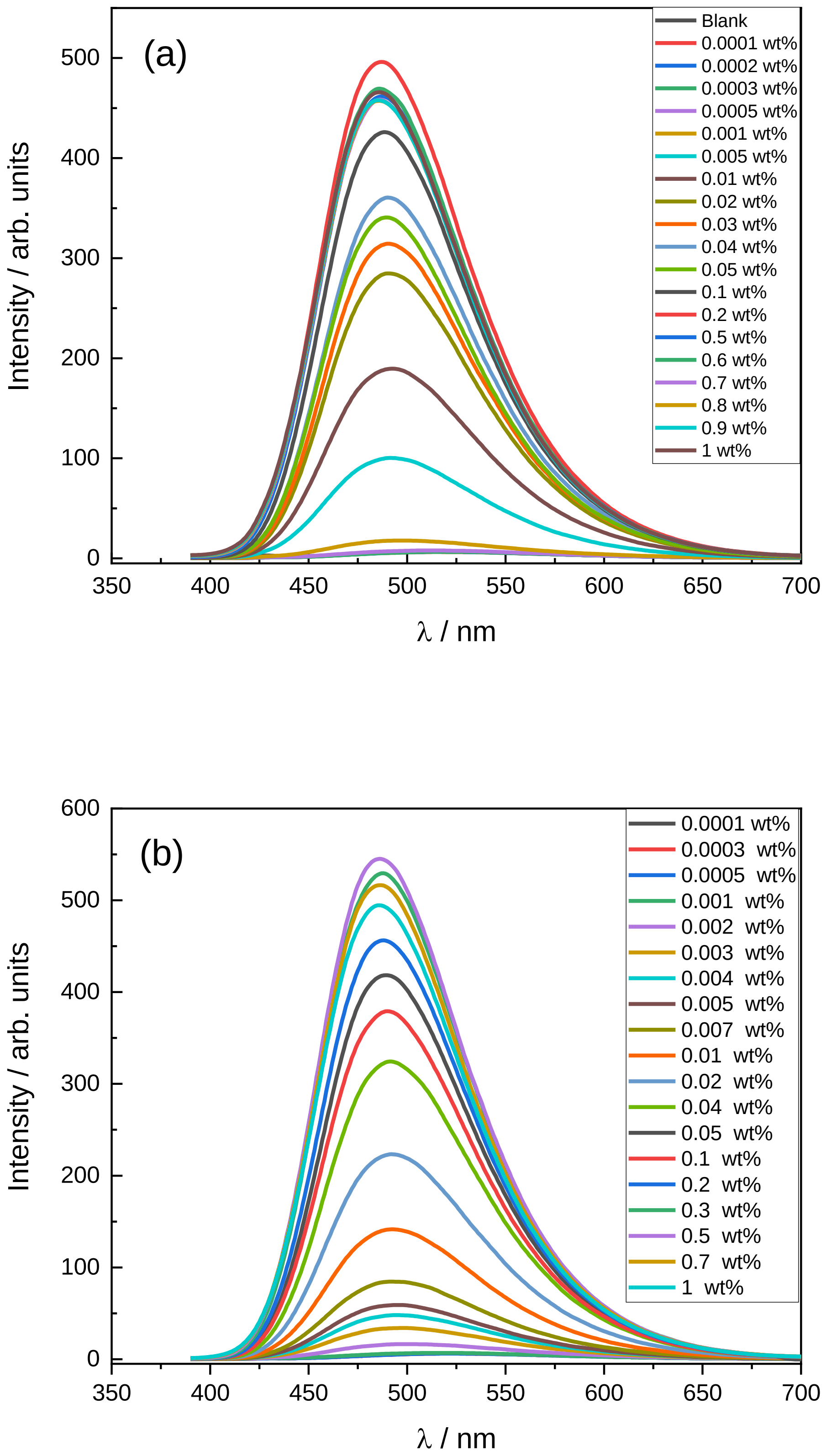


**Figure S1.** ANS Fluorescence spectra for (a) $C_{16}$-$CSK_4RGDS$, (b) $C_{16}$-$CSK_4GRDS$.

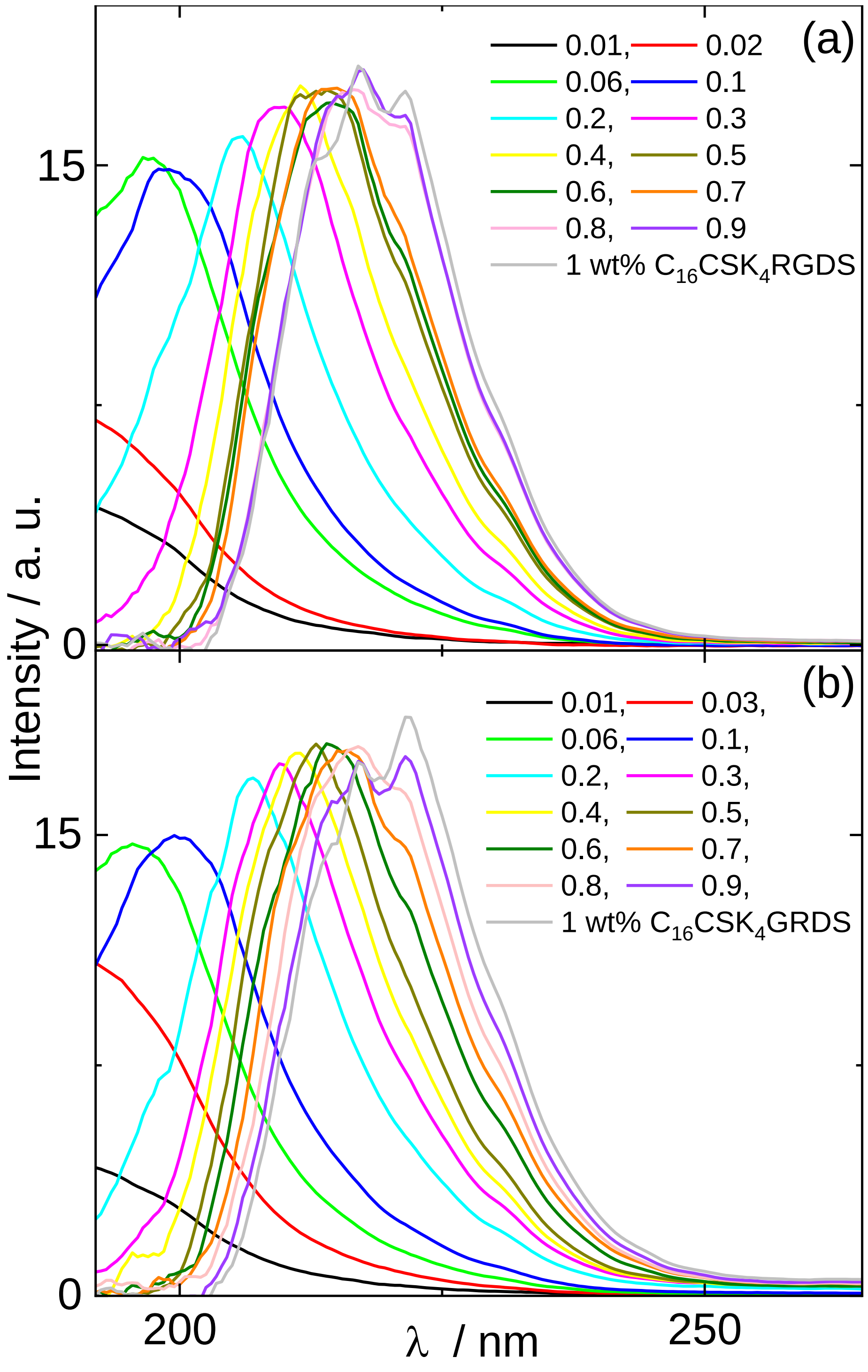


**Figure S2.** UV/vis spectra for (a) $C_{16}$-$CSK_4$RGDS, (b) $C_{16}$-$CSK_4$GRDS.

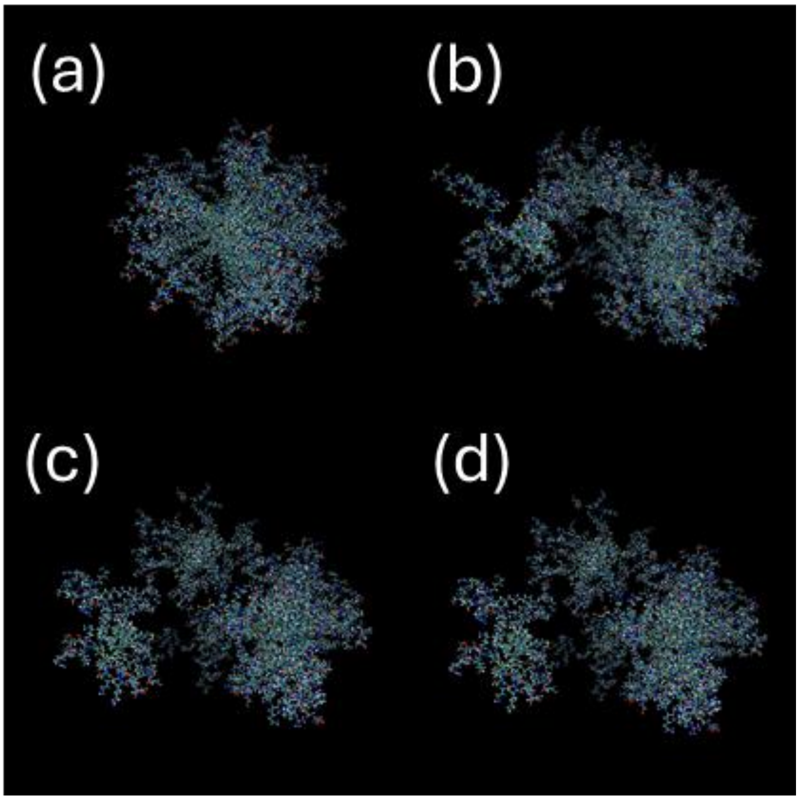


**Figure S3.** Snapshots of micelles of $C_{16}$-$CSK_4RGDS$ with *p* = 60 (and charge +3) from MD simulations at time points (a) t = 0 (b) t =2.5 ns, (c) t = 6.09 ns, (d) t = 10 ns.

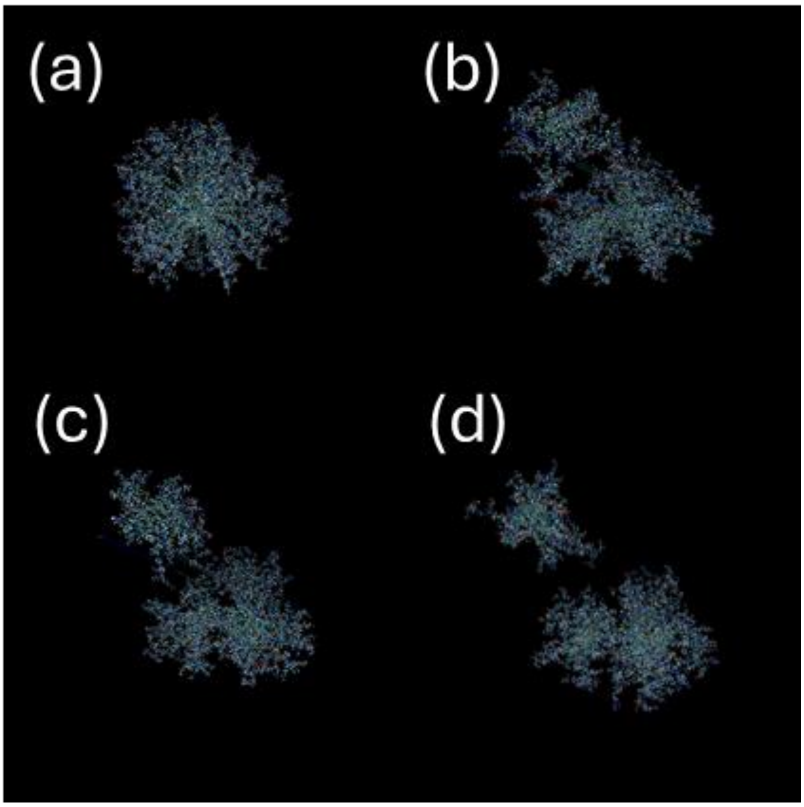


**Figure S4.** Snapshots of micelles of $C_{16}$-$CSK_4GRDS$ with $p$ = 60 (and charge +3) from MD simulations at time points (a) t = 0 (b) t =2.5 ns, (c) t = 4.59 ns, (d) t = 10 ns.

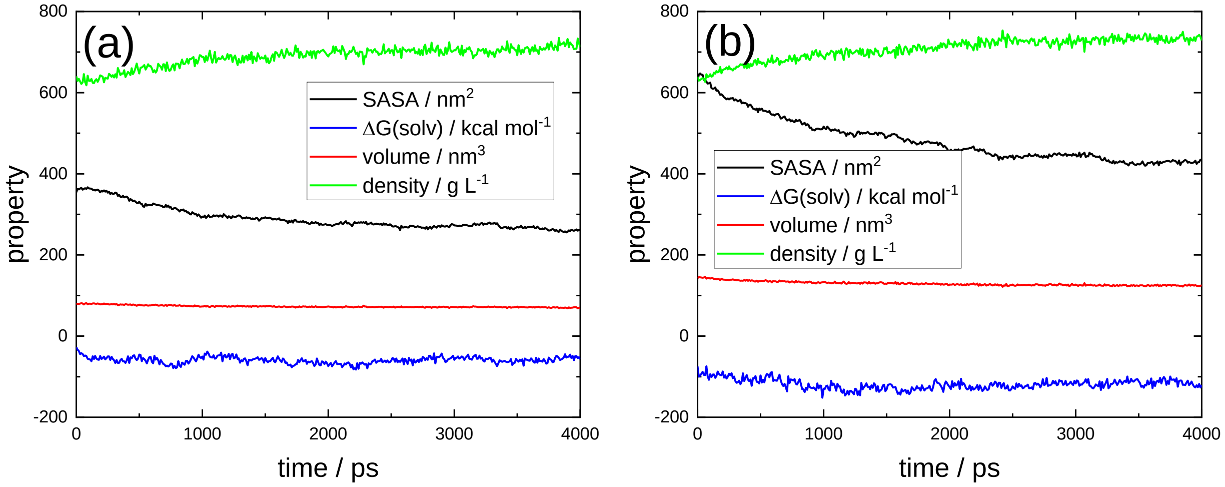


**Figure S5.** SASA and solvation-related properties calculated from MD simulations (a) $C_{16}$-$CSK_4$RGDS (*p* = 22, charge +2 per molecule) (b) $C_{16}$-$CSK_4$GRDS (*p* = 40, charge +2 per molecule)

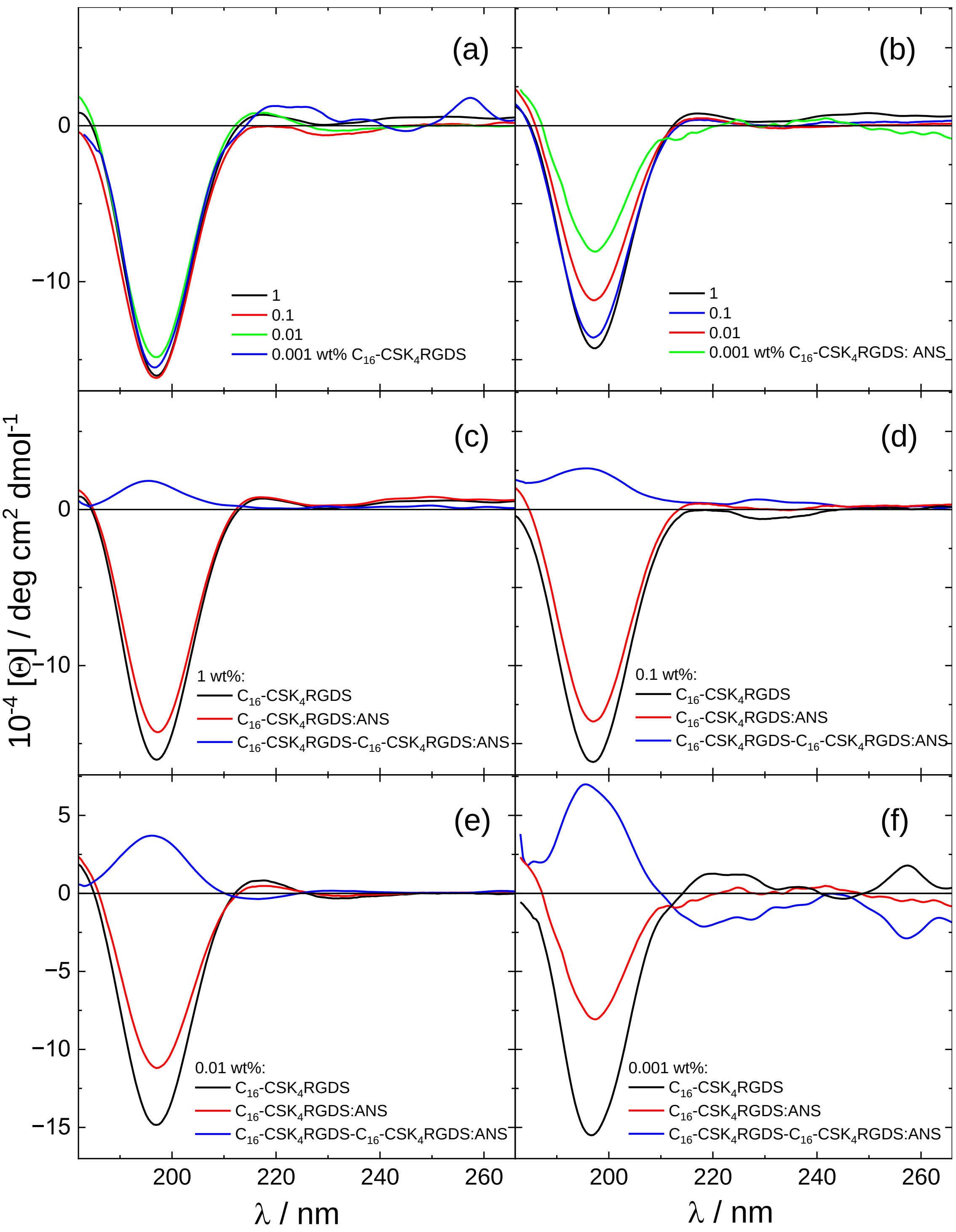


**Figure S6**. Difference CD spectra to probe ANS binding, under conditions indicated for $C_{16}$-$CSK_4RGDS$. Spectra for (a) $C_{16}$-$CSK_4RGDS$ at concentrations indicated (same data as Fig.2a), (b) $C_{16}$-$CSK_4RGDS$ with 0.002 wt% ANS at lipopeptide concentrations indicated. Spectra for (c) 1, (d) 0.1, (e) 0.01, and (f) 0.001 wt% $C_{16}$-$CSK_4RGDS$ with and without ANS and difference.

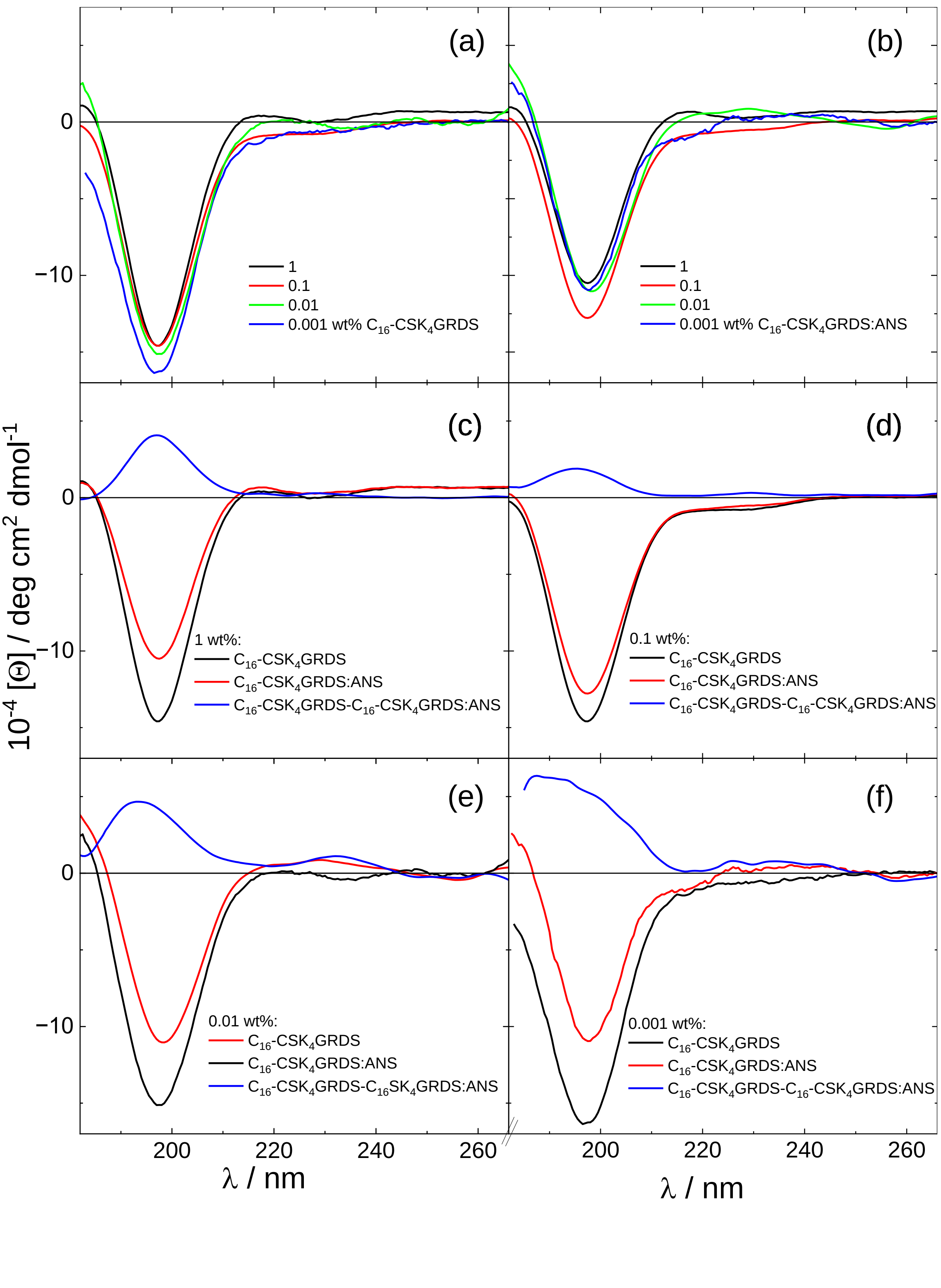


**Figure S7**. Difference CD spectra to probe ANS binding, under conditions indicated for $C_{16}$-$CSK_4GRDS$. Spectra for (a) $C_{16}$-$CSK_4GRDS$ at concentrations indicated (same data as Fig.2a), (b) $C_{16}$-$CSK_4GRDS$ with 0.002 wt% ANS at lipopeptide concentrations indicated. Spectra for (c) 1, (d) 0.1, (e) 0.01, and (f) 0.001 wt% $C_{16}$-$CSK_4GRDS$ with and without ANS and difference.

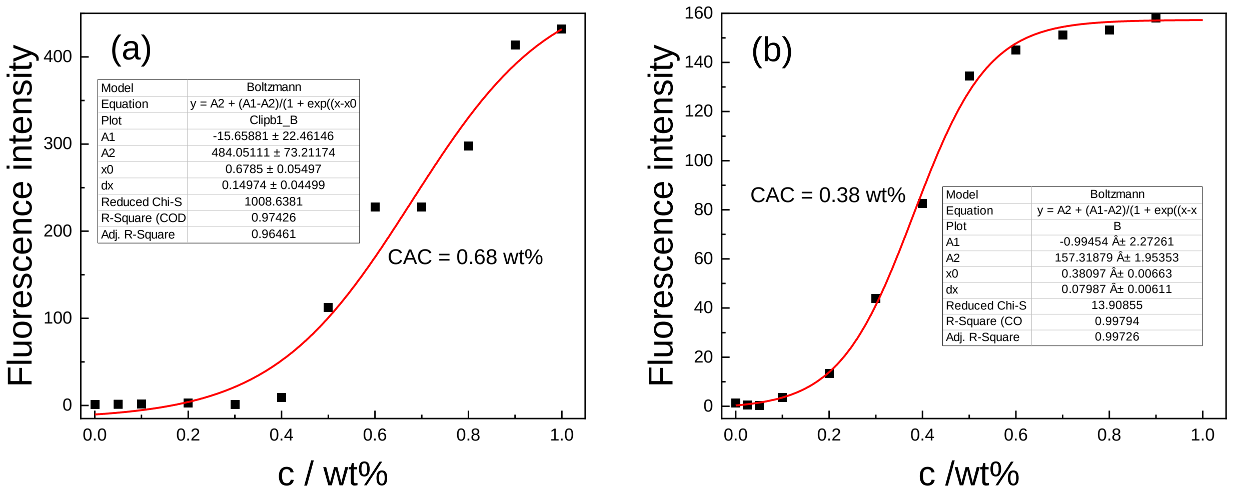


**Figure S8**. Fluorescence probe assays of CMC using Nile red. The fluorescence intensity at $\lambda_{em}$ = 642 nm is plotted as a function of lipopeptide concentration. simulations (a) $C_{16}$-$CSK_4RGDS$, (b) $C_{16}$-$CSK_4GRDS$. The data were fitted using Boltzmann-type sigmoidal functions[1-2] and the midpoint (x0 in the inset tables) defines the CMC.

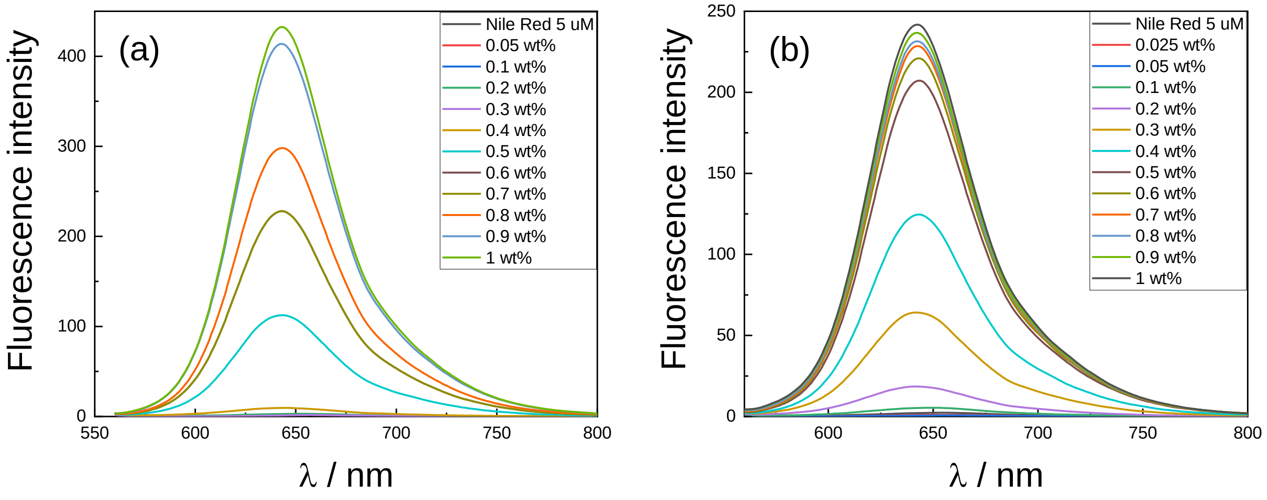


**Figure S9**. Nile red fluorescence spectra for (a) $C_{16}$-CSK$_4$RGDS, (b) $C_{16}$-CSK$_4$GRDS.

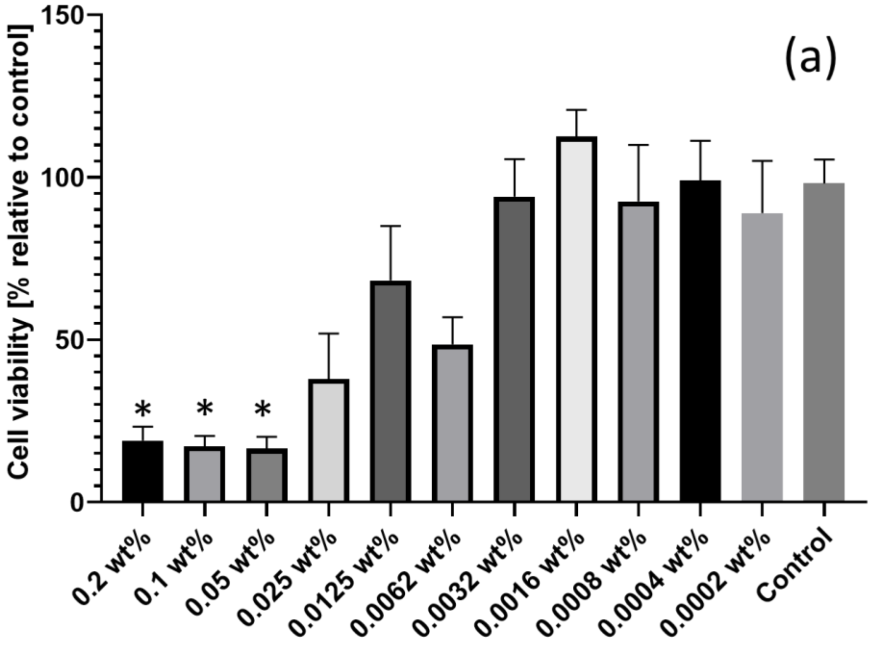


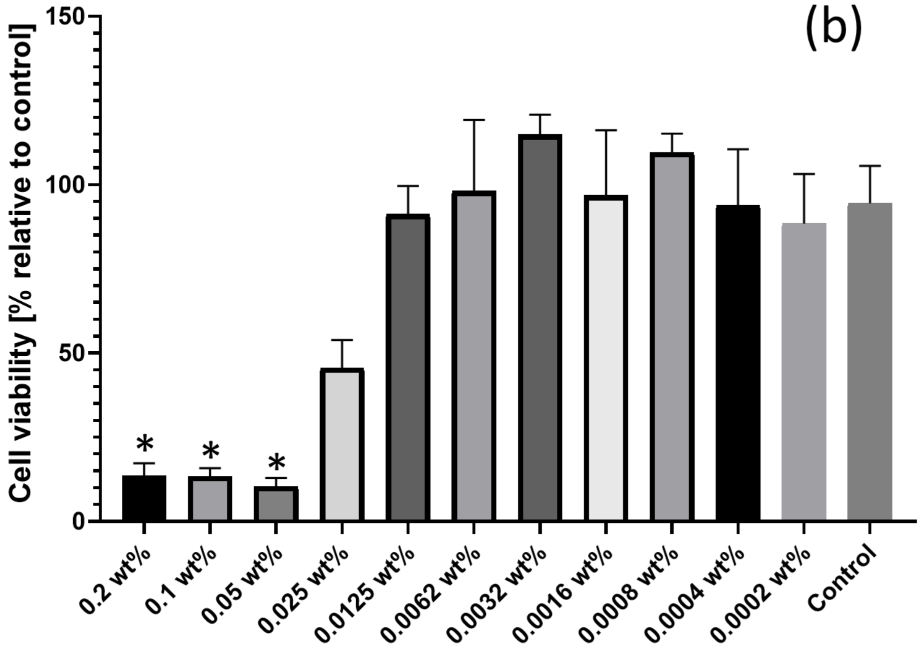


**Figure S10.** Cell viability after 72h for C2C12 myoblasts from MTT assays for (a) $C_{16}$-$CSK_4RGDS$, (b) $C_{16}$-$CSK_4GRDS$. * $p \leq 0.05$.

**Table S1**. SAXS data fitted parameters. SAXS data fitted using SASfit,[3-4] using a core-shell sphere form factor model and hard sphere structure factor for 2 wt% solutions, and for $C_{16}$-$CSK_4RGDS$ an additional form factor term to account for monomers represented as generalized Gaussian coils.

| **Parameter** | **1 wt% $C_{16}$-$CSK_4GRDS$** | **2 wt% $C_{16}$-$CSK_4GRDS$** | **1 wt% $C_{16}$-$CSK_4RGDS$** | **2 wt% $C_{16}$-$CSK_4RGDS$** |
|---|---|---|---|---|
| $R_o$ / Å | 30.4 | 29.0 | 30.0 | 30.0[a] |
| $\Delta R$ / Å | 2.3 | 5.3 | 4.2 | 3.1 |
| $R_i$ / Å | 15.8 | 14.0 | 14.0 | 14.0 [a] |
| $\mu$ | -0.125 | -0.359 | 0.056 | -0.651 |
| $\eta$ / cm$^{-1}$ | $3.29 \times 10^{-6}$ | $5.66 \times 10^{-6}$ | $2.15 \times 10^{-6}$ | $4.22 \times 10^{-6}$ |
| $R_g$ / Å | - | - | 10.0 | 30.0 |
| $\nu$ | - | - | 0.18 | 0.18 [a] |
| $I_0$ / cm$^{-1}$ | - | - | $4.22 \times 10^{-3}$ | $1.02 \times 10^{-1}$ |
| $R_{HS}$ / Å | - | 59.7 | - | 58.0 |
| $\phi_p$ | - | 0.32 | - | 0.45 |
| BG / cm$^{-1}$ | $2.9 \times 10^{-3}$ | $4.1 \times 10^{-3}$ | $2.1 \times 10^{-3}$ | $4.5 \times 10^{-4}$ |

Parameters: **Core-Shell Sphere Form Factor** - $R_o$, outer radius (Gaussian polydispersity $\Delta R$); $R_i$, inner core radius; $\mu$, scattering contrast of inner core (relative to shell); $\eta$, scattering contrast of shell; **Sphere Structure Factor -** $R_{HS}$, hard sphere radius; $\phi_p$ volume fraction; **Generalized Gaussian Coil** - $R_g$, radius of gyration; $\nu$, Flory exponent; $I_0$, intensity; **Background** - BG

**[a]** Parameters fixed to same values as for the 1 wt% solution

## References


(1) Carpena, P.; Aguiar, J.; Bernaola-Galván, P.; Ruiz, C. C., Problems associated with the treatment of conductivity-concentration data in surfactant solutions:: Simulations and experiments. *Langmuir* **2002,** *18* (16), 6054-6058.

(2) Al-Soufi, W.; Novo, M., A Surfactant Concentration Model for the Systematic Determination of the Critical Micellar Concentration and the Transition Width. *Molecules* **2021,** *26* (17), 5339.

(3) Bressler, I.; Kohlbrecher, J.; Thünemann, A. F., SASfit: a tool for small-angle scattering data analysis using a library of analytical expressions. *J. Appl. Cryst.* **2015,** *48*, 1587-1598.

(4) Kohlbrecher, J.; Bressler, I., Updates in SASfit for fitting analytical expressions and numerical models to small-angle scattering patterns. *J. Appl. Cryst.* **2022,** *55*, 1677-1688.
